# PARTIAL REPROGRAMMING OF THE AGING NEUROGENIC NICHE

**DOI:** 10.1093/geroni/igae098.0870

**Published:** 2024-12-31

**Authors:** Lucy Xu, Julliana Ramirez-Matias, Max Hauptschein, Eric Sun, Judith Lunger, Matthew Buckley, Anne Brunet

**Affiliations:** Harvard Medical School, Boston, Massachusetts, United States; Stanford University, Stanford, California, United States; Stanford University, Stanford, California, United States; Stanford University, Stanford, California, United States; Stanford University, Stanford, California, United States; Stanford University, Stanford, California, United States; Stanford School of Medicine, Stanford, California, United States

## Abstract

Reprogramming of somatic cells by constant expression of the Yamanaka factors (Oct4, Sox2, Klf4, c-Myc) results in pluripotent stem cells and erases many hallmarks of aging in vitro. Partial reprogramming, or expression of the Yamanaka factors in a time- or cell-restricted manner, improves function in a variety of cell types from aging mice without loss of cell identity. But the impact of partial reprogramming on the aged brain has remained largely unknown. We examined how partial reprogramming affects the subventricular zone (SVZ), a conserved neurogenic niche in mammalian brains. SVZ neural stem cells give rise to newborn neurons and astrocytes throughout adulthood, but the process of neurogenesis declines with age, with a drop in neuronal precursor cells (neuroblasts) and negative impacts on olfactory discrimination and learning. Using single-cell RNA-sequencing, we find that partial reprogramming of the whole body in old mice restores the proportion of neuroblasts - cells committed to give rise to new neurons - in the neurogenic niche. Several molecular signatures of aging are also reverted by partial reprogramming. Interestingly, partial reprogramming in the SVZ neurogenic niche itself increases the proportion of neuroblasts and neural stem cells in old mice. Partial reprogramming of old neural stem cells in culture improves transcriptomic signatures of aging and boosts neuroblast and neuron production during differentiation, suggesting cell-autonomous effects. We find that partial reprogramming can rejuvenate aspects of the neural stem cell lineage in old brains, raising the possibility that this intervention could be used to counter brain decline in old individuals.

